# Quantitation of Risk Reduction of *E. coli* Transmission after Using Antimicrobial Hand Soap

**DOI:** 10.3390/pathogens9100778

**Published:** 2020-09-23

**Authors:** Umesh Adhikari, Elaheh Esfahanian, Jade Mitchell, Duane Charbonneau, Xiangyu Song, Yang Lu

**Affiliations:** 1 Department of Biosystem and Agricultural Engineering, Michigan State University, East Lansing, MI 48823, USA; adhika12@msu.edu (U.A.); e.esfahanian86@gmail.com (E.E.); 2 Procter & Gamble Company, Cincinnati, OH 45040, USA; charbonneau.dl@pg.com; 3 Procter & Gamble Technology (Beijing) Co., Ltd., Beijing 101312, China; song.xi@pg.com (X.S.); lu.yg@pg.com (Y.L.)

**Keywords:** quantitative microbial risk assessment, QMRA, foodborne illness, *Escherichia coli*, fomites, handwashing, antimicrobial soap

## Abstract

Handwashing with soap is an effective and economical means to reduce the likelihood of *Escherichia coli* infection from indirect contact with contaminated surfaces during food preparation. The purpose of this study was to conduct a quantitative microbial risk assessment (QMRA) to evaluate the risk of infection from indirect contact with fomites contaminated with *E. coli* after hand washing with antimicrobial hand soaps. A Monte Carlo simulation was done with a total of 10,000 simulations to compare the effectiveness of two antimicrobial and one control (non-antimicrobial) bar soaps in reducing the exposure and infection risk compared to no hand washing. The numbers of *E. coli* on several fomites commonly found in household kitchens, as well as the transfer rates between fomites and onto fingertips, were collected from the literature and experimental data. The sponsor company provided the *E. coli* survival on hands after washing with antimicrobial and control soaps. A number of scenarios were evaluated at two different exposure doses (high and low). Exposure scenarios included transfer of *E. coli* between meat-to-cutting board surface-to-hands, meat-to-knife surface-to-hands, and from a countertop surface-to-hands, kitchen sponge-to-hands, hand towel-to-hands, and dishcloth-to-hands. Results showed that the risks of illness after washing with the control soap was reduced approximately 5-fold compared to no handwashing. Washing with antimicrobial soap reduced the risk of *E. coli* infection by an average of about 40-fold compared with no handwashing. The antimicrobial soaps ranged from 3 to 32 times more effective than the non-antimicrobial soap, depending on the specific exposure scenario. Importance: The Centers for Disease Control and Prevention indicate the yearly incidence rate of Shiga Toxin producing *E. coli* infections is about 1.7/100,000, with about 10% of cases leading to life-threatening hemolytic uremic syndrome and 3–5% leading to death. Our findings confirm handwashing with soap reduces the risks associated with indirect transmission of *E. coli* infection from contact with fomites during food preparation. Further, in these exposure scenarios, antimicrobial soaps were more effective overall than the non-antimicrobial soap in reducing exposure to *E. coli* and risk of infection.

## 1. Introduction

Foodborne illness can be caused by a wide variety of pathogens. In 2011, using data from active and passive surveillance from 2000–2008, Ref. [1] reported that 31 major pathogens cause an estimated 9.4 million episodes of foodborne illness in the United States each year, with over 55,000 hospitalizations and over 1300 deaths. Approximately a third of these foodborne illnesses are caused by bacteria. *Escherichia coli* is one of the pathogens that can lead to foodborne disease. *E. coli* is a large and diverse group of gram-negative bacteria that commonly inhabit the intestines of healthy humans. Many strains are harmless; however, some are pathogenic, and can pose serious health threats including diarrhea and gastrointestinal illness, urinary tract infections, respiratory illness, and pneumonia. In 2016, the Centers for Disease Control and Prevention (CDC), National Enteric Disease Surveillance: Shiga Toxin-producing Escherichia coli (STEC) Annual Report indicated a yearly incidence of 5441 cases of culture-confirmed Shiga toxin-producing *E. coli* (STEC) infections, including 2323 O157 and 3104 non-O157 cases. Additionally, 1574 STEC reports were submitted with unknown serogroup [2].

Among the pathogenic strains of *E. coli*, the one commonly associated with foodborne outbreaks is the Shiga toxin-producing pathotype, especially *E. coli* O157. Foodborne transmission is common for *E. coli* O157. Scallan [3] reported there are an estimated 63,000 cases of domestically acquired foodborne illness due to *E. coli* O157 annually, resulting in an estimated 2000 hospitalizations and 10 deaths. Keithlin [4] reported that gastrointestinal disease caused by *E. coli* O157 infection can lead to chronic conditions such as reactive arthritis and hemolytic uremic syndrome. *Escherichia coli* O157 can be transmitted directly through the ingestion of contaminated food or water, and indirectly on hands that have been in contact with contaminated surfaces (surface-to-hand-to-face).

Fomites refer to non-living materials that humans contact every day including door handles, paper, cellphones, and sinks [5]. They also refer to surfaces used in the preparation of food and clean-up such as, cutting boards, knives, and kitchen towels. Enteric bacteria such as *E. coli* are commonly found on kitchen surfaces, resulting in opportunities for indirect transmission.

Alcohols are generally considered very effective antimicrobials [6,7]. Isopropanol is a reference agent for testing the efficacy of hygienic hand disinfection in Europe (European standard EN 1500) [6]. A 60% concentration using a reference treatment of two 3-mL doses for a total of 60 s produces a mean reduction *E. coli* of 4.6 log_10_ units (about 40,000) on hands artificially contaminated with the bacterium [6]. Ansari [8] investigated the efficacy of handwashing agents in removing *E. coli* from the finger pads of adult volunteers. Results indicated that 70% isopropanol or ethanol produced >98% reduction in *E. coli* after 10 s of exposure. However, bactericidal activity is observed at concentrations as low as 30% for ethanol, propanol, and isopropanol [6].

The effectiveness of handwashing with soap in reducing illnesses such as diarrhea and respiratory infection has been demonstrated in epidemiological studies [9,10]. However, the magnitude of the reduction has been more difficult to define. Estimates range from 20% to 47% [10,11,12]. In a review of worldwide handwashing practices, Freeman [9] reviewed 42 published reports on handwashing prevalence worldwide based on direct observation. The authors used a meta-regression technique to determine that handwashing reduces the risk of diarrheal disease by 40%. However, experience has shown that direct observation can markedly increase handwashing practices. When the authors included an adjustment to estimate the bias introduced by observation risk reduction dropped to 23%.

Quantitative microbial risk assessment (QMRA) is a process for quantitatively estimating the human health risk associated with a specific pathogen through an environmental exposure [13,14,15,16,17]. A recent publication by Membré and Boué [18] provided guidance on developing QMRA to evaluate different risk reduction measures and operational procedures in the food industry. Franz [19] incorporated pathogen growth modeling and storage temperature into a QMRA on risks associated with leafy greens in restaurant salad bars. This approach can also be used to develop microbial reduction targets for the sanitization of surfaces in places accessible to the public, such as schools, restaurants, and nursing homes [5]. We used a similar approach to evaluate the effects of treating porous surfaces with an antimicrobial spray on the risk of viral infection [20].

In this study, QMRA was used to compare the effectiveness of handwashing with two proprietary antimicrobial bar soaps versus a non-antimicrobial bar soap (control) in reducing the potential risks from oral exposure to *E. coli* resulting from transfer during food preparation.

## 2. Materials and Methods

### 2.1. Data Collection

The bacteria survival study was conducted following the ASTM Standard (ASTM Standard E2752, 2010) with slight modifications. This was a randomized, double-blind study. It consisted of a washout period that lasted 7 to 14 days avoiding contact with any product with anti-bacteria efficacy, followed by a 3-day washing period. During the washing period, the hands were washed following standard wash procedure 3 times a day, with at least 1 h interval. Three groups of subjects, 20 in each group, were used—2 test bars (antimicrobial #1, antimicrobial #2) and 1 placebo bar for hand wash. Four hours after the ninth wash, one area on palm of one hand was marked off, inoculated with *Escherichia coli* (ATCC 10536), and occluded with an occlusive chamber patch. About 5 h after occlusion, the patch was removed, and the bacteria were harvested using a scrub technique. The harvested bacteria were diluted, plated, and incubated for about 24 h before counting and calculations.

The test bars were mainly composed of sodium fatty acid, with 25–30% soluble soap, pH > 10, and water content 9–15%. The test bars of antimicrobial #1 and antimicrobial #2 contained various levels of zinc pyrithione (ZnPT) as the key antibacterial active.

### 2.2. Exposure Assessment

**Figure 1:**
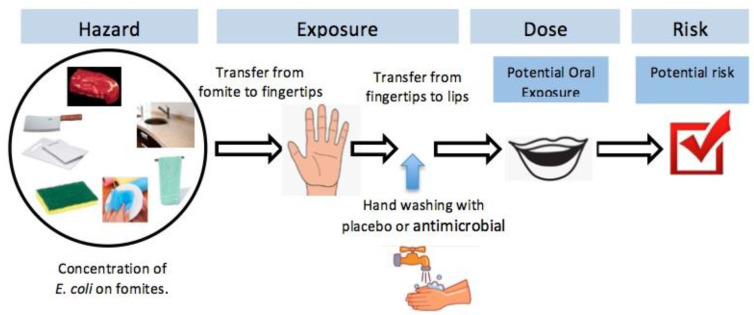
The reported *Escherichia coli* concentrations on various fomites, along with the transfer rates to secondary surfaces to hands, and from hands to lips were used in a Monte Carlo analysis to determine the resulting potential oral exposure dose and potential risk of illness with and without hand washing.

In this study, the potential oral exposure to *E. coli* from transfer during food preparation was considered. Figure 1 shows the risk assessment framework. The reported *E. coli* concentrations on various fomites were used as a starting point, along with the transfer rates to secondary surfaces. Fomites considered in this assessment included: Cutting board (i.e., high density polyethylene or HDPE), knife (i.e., stainless steel), meat, kitchen countertop (i.e., granite), hand towel and dishcloth (i.e., 100% cotton), and kitchen sponge. Table 1a shows the *E. coli* concentrations reported on different primary surfaces, and transfer rates to secondary surface along with the types of distribution. During the experiment, the fomites were inoculated with two levels of *E. coli*: High dose (6 log_10_ CFU/sq. cm) and low dose (3 log_10_ CFU/sq. cm) [21]. The transfer rates from various fomites to fingertips are given in Table 1b. The bacteria survival study was conducted following the ASTM Standard (ASTM Standard E2752, 2010). The percentage survival of bacteria on the hands after washing with each of the three bars is given in Table 1c, along with the fingertip-to-mouth transfer rate. Under each scenario, the exposure dose was estimated by multiplying the *E. coli* at the fingertip with fingertip-to-mouth transfer rate.

### 2.3. Dose-Response Assessment

A dose-response model describes the relationship between the exposure dose and the host response at a given dose. Mathematical functions are used to calculate the likelihood of an adverse health outcome. A variety of *E. coli* dose-response models have been developed under different experimental conditions [29]. To capture the range of variability, three models were selected for evaluation (shown in Table 2) including: A recommended best fit model for *E. coli* (using EIEC 1624) with the endpoint being the presence of *E. coli* in the stool, the highest median infectious dose (ID_50_) model (using ETEC B7A) with the endpoint being mild to severe diarrhea, and the lowest ID_50_ model (using ETEC B7A) with the endpoint being the presence of *E. coli* in the stool. Best fit model for all of the experiments were the beta-Poisson model as shown in Equation (1).

(1) $$p\left(response\right)=1-{\left[1+dose\frac{\left(2^{\frac{1}{\alpha }}-1\right)}{N_{50}}\right]}^{-\alpha }$$

where, *p(response)* is the probability of infection or illness, *dose* is the exposure dose, *α* is the slope parameter for beta-Poisson model, and *N*_50_ is the median infectious dose.

### 2.4. Risk Characterization

In order to estimate the risk of *E. coli* illnesses including variability and uncertainty across the modeled exposure pathway in Figure 1, a Monte Carlo analysis was conducted using the Crystal Ball^®^ program (Oracle, Redwood Shores, CA, USA). Probability distributions for each uncertain model parameter were developed as described in Table 1. A total of 10,000 simulations were run to estimate the distribution of exposure dose first then the risk of infection using each dose-response model per established methods [14]. Two results are presented below to describe the effects of handwashing on the risks of illness from handling contaminated fomites: (1) The potential oral exposure dose, and (2) the resulting potential risk.

## 3. Results

### 3.1. Effect of Antimicrobials on the Potential Exposure Dose

The potential exposure dose was determined after transfer from an initial surface-to-hands-to-lips (Figure 1). In some cases, a secondary surface was also included. Results of the Monte Carlo analysis indicated that washing hands with either the control bar soap, or the antimicrobial #1 or #2, reduced the potential exposure dose to *E. coli* from contaminated fomites. As shown in Table 3a, transfer from meat preparation surfaces without subsequent washing resulted in potential exposures ranging from 2.4 × 10^1^ CFU (cutting board-to-meat, low dose) to 1.8 × 10^5^ CFU (meat-to-knife, high dose). If the hands were washed with the control bar soap, potential exposure was reduced to 4.3 × 10^0^ CFU and 2.4 × 10^4^ CFU, respectively. Washing with the antimicrobial soaps resulted in greater reductions in the potential exposures. The exposure from cutting board-to-meat, low dose, resulted in 7.6 × 10^−1^ CFU and 1.1 × 10^0^ CFU, after washing with antimicrobial #1 and #2, respectively. Exposures from meat-to-knife, high dose, were 2.0 × 10^3^ CFU and 1.3 × 10^3^ CFU. Similar reductions in potential exposure were observed with other typical kitchen surfaces (Table 3a).

Median values for the exposure doses were used to calculate a reduction factor in exposure for the handwashing protocols compared to the exposure with no handwashing (Table 3b). Overall, handwashing with control soap reduced the potential exposure by a factor of ≥5.4 compared to no handwashing, whereas washing with either antimicrobial #1 or #2 reduced potential exposure by a factor of ≥20.

It is notable that exposures from some surfaces such as meat-to-knife (high and low dose) were reduced to a greater extent than exposures from the other surfaces. Washing with the control bar reduced the exposure by a factor of ≥7 in this instance, washing with antimicrobial #1 resulted in a reduction factor of ≥83, and antimicrobial #2 resulted in a reduction factor of ≥137. When the surface was a hand towel, exposure was reduced by a factor of 5.4 after using the control, and by a factor of over 780 after using antimicrobial #1. An exposure reduction factor could not be calculated for antimicrobial #2 since the median exposure after using this soap product was reduced to zero.

**Figure 2:**
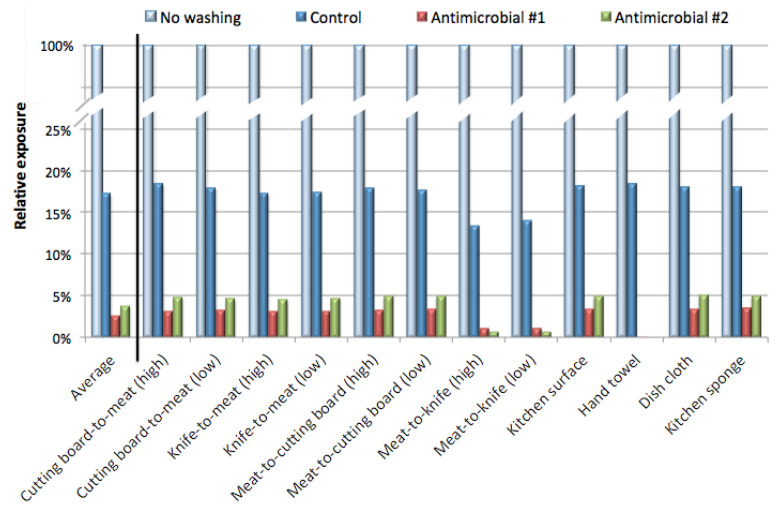
The potential exposure doses with and without hand washing (from Table 3) were used to calculate the reduction in exposure with each hand washing practice (washing with the control bar, antimicrobial #1, or antimicrobial #2). Results were expressed as a percentage of the exposure observed with the same experimental conditions, but without hand washing. The average of all scenarios is presented first, along with the exposure reduction for each scenario.

The reduction in exposure is plotted in Figure 2, showing the average reduction for all scenarios, and the reduction for individual scenarios. Washing with the control bar reduced the median number of *E. coli* transferred from contaminated hands to the lip to an average of about 17% of the *E. coli* exposure that would have resulted from no washing. Washing with either one of the antimicrobial bars reduced the average exposure to <4% compared to no washing. In this study washing with the antimicrobials was over 4-fold more effective in reducing exposure than washing with the control soap.

### 3.2. Effect of Antimicrobials on Risk

Table 4 presents results of the potential risk analysis using the three different dose response models shown in Table 2: The recommended best fit model, and the high infectious dose and low infectious dose models. Table 4a shows that, using the recommended dose response model, the estimated risk of infection from contaminated meat preparation surfaces with no hand washing ranges from 1.6 × 10^−4^ (cutting board-to-meat, low dose) to 2.8 × 10^−1^ (meat-to-knife, high dose). By washing with the control bar soap, the estimated risk dropped to 2.7 × 10^−5^ and 1.1 × 10^−1^, respectively, or about a factor of 5.9. Washing with the antimicrobial soaps resulted in greater risk reductions. The risk from cutting board-to-meat, low dose, was estimated to be 4.8 × 10^−6^ and 7.2 × 10^−6^ after washing with antimicrobials #1 and #2, respectively. For meat-to-knife, high dose, the risk was 1.2 × 10^−2^ and 7.9 × 10^−3^. Similar reductions in risks were observed with other typical kitchen surfaces (Table 4a). 

In Table 4b median values for risk from Table 4a were used to calculate a reduction factor for each of the three handwashing protocols. Using the recommended best fit model, washing with the control bar reduced the risk by an average of about 5-fold compared to the risk calculation with no washing. However, washing with antimicrobials #1 and #2 reduced the risk by average factors of 45 and 44, respectively. The risk reduction factor with the antimicrobial soaps varied considerably with different exposure scenarios. In particular, exposures from the meat-to-knife (low dose) and from the hand towel resulted in very high-risk reduction factors compared to “no washing”.

As mentioned above, Table 4b shows the risk reduction factor for the recommended best fit model. The other two dose response models (risk with high and low infectious dose) produced similar results.

**Figure 3:**
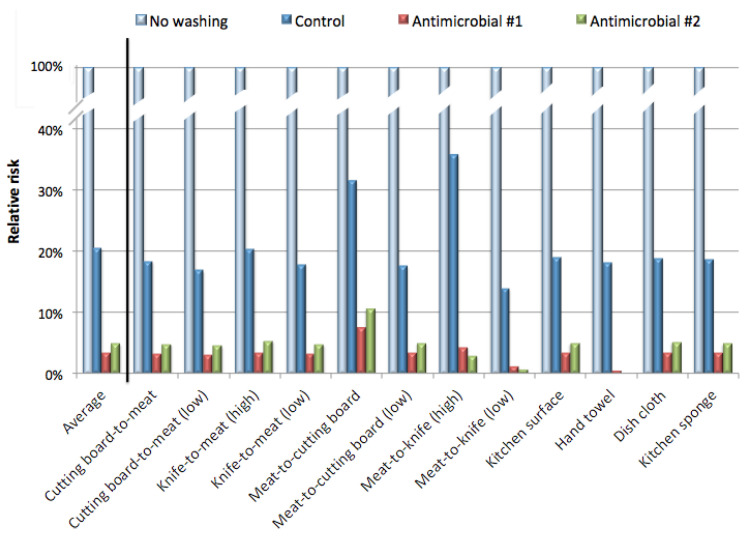
The estimated risks with and without hand washing (from Table 4) were used to calculate the risk reduction factor with each hand washing practice (washing with the control bar, antimicrobial #1, or antimicrobial #2). This was determined by dividing the median risk from “no washing” by the median risk after washing with each of three soaps. Results from the recommended dose-response model are presented, with the average of all scenarios shown first, along with the risk reduction factor for each scenario.

Figure 3 illustrates the reduction in risk determined for each exposure scenario from the recommended best fit model. Washing with the control bar soap reduced the estimated risk by an average of about 20% compared to “no washing”. In contrast, washing with antimicrobials #1 and #2 reduced the risk by an average of about 3%.

## 4. Discussion

Handwashing with soap is universally accepted as an effective means of reducing the risk of transmitting disease [11,12,30]. However, studies conducted to demonstrate superior performance by antimicrobial soaps compared to non-antimicrobial soaps have had mixed success. Kampf and Kramer [6] reported that antimicrobial agents in soaps can provide more effective removal of microorganisms provided the duration of the washing process is long enough. A one-minute hand wash could reduce *E. coli* between 0.5–2.8 log_10_ units (or about 3–600-fold). In a study conducted by Fuls et al., [31] washing with antimicrobial soap was found to reduce the bacterial load of both *Shigella flexneri* and *E. coli* to a greater extent than a non-antimicrobial soap. The authors concluded the effectiveness of antimicrobial soaps could be improved with longer wash time and greater soap volume. In a recent study reported by Pérez–Garza et al. [32], antimicrobial soaps removed levels of *E. coli* similar to those removed by non-antimicrobial soap on hands contaminated with *E. coli* at 10^3^ CFU/g. However, when hands were contaminated with *E. coli* at 10^6^ CFU/g, the antimicrobial soap was more effective at removing *E. coli*.

In contrast to data supporting the superiority of antimicrobial hand soaps, other authors have concluded the opposite. In a review of published experimental studies, de Witt Huberts et al. [33] concluded that washing with antimicrobial products was no more effective than using non-antimicrobial soaps in removing pathogens from hands. Recently, Jensen et al. [34] reported results comparing an antimicrobial soap formulation containing chloroxylenol versus a non-antimicrobial soap. The two products were not significantly different at removing *E. coli* using lather times of 10, 20, and 40 s.

Gibson et al. [28] used a QMRA approach to examine the effectiveness of different soap formulations on the probability of *Shigella* infection after diaper changing. These investigators reported a reduction in bacterial loads with the use of either non-antimicrobial or antimicrobial soaps. The mean log_10_ reduction in the number of organisms was 2.56 (about 360-fold) with control soap, 2.61 (about 400-fold) with a chlorhexidine-containing soap, and 2.91 (about 800-fold) with a triclosan-containing soap.

Ryan et al. [5] published reports on contaminated hard, nonporous fomites, and transfer to hands of pathogenic or surrogate organisms was conducted with the goal of summarizing the average concentrations and types of microbes found on such surfaces. The authors then conducted a QMRA to determine the effects of sanitizing the surfaces on the risks of infection. The QMRA analysis suggests that a reduction in bacterial numbers on a fomite by 99% (2 log_10_ units) will likely reduce the risk of infection from a single contact to less than 1 in 1 million.

In our QMRA analysis we found that handwashing with a control (non-antimicrobial) soap reduced the exposure by a factor of 5.4–7.5 compared to no washing, and washing with the antimicrobial soaps reduced exposure by a factor of 20–781 (Table 3b).

The reduction in exposure and risk varied considerably with different exposure scenarios and different fomites (Table 3b and Table 4b, respectively). This variation can be only partially explained by the input values for *E. coli* concentration on the surfaces and transfer rates (Table 1). Transfer of bacteria to fingertips is known to vary based on the nature of the fomite surface. Nonporous fomites have a much higher transfer efficiency than porous fomites. Rusin et al. [15] used a pooled culture of a gram-positive bacterium (*Micrococcus luteus*), a gram-negative bacterium (*Serratia rubidea*), and phage PRD-1 to evaluate the fomite-to-hand transfer efficiencies from several common materials. Transfer efficiencies ranging from 27.6% to 65.8% from the nonporous surfaces (phone receiver and faucet), and <0.13% from porous surfaces (dishcloth, sponge, 100% cotton and 50:50 cotton/polyester). Interestingly, carrot and hamburger showed transfer efficiencies similar to nonporous surfaces at 0.12–0.35% and <0.01–0.06%, respectively. A similar result was obtained by Lopez et al. [27]. These investigators used a variety of organisms including gram-negative and gram-positive bacteria, spore-forming bacteria, and viruses to investigate the transfer efficiencies from fomite-to-finger. Nonporous surfaces such as granite, stainless steel, and glass had a fomite-to-finger transfer efficiency of up to 57% under low humidity conditions, and 79.5% under high humidity conditions. Porous surfaces such as cotton, polyester, and paper currency had transfer efficiencies of <6.8% in low humidity and <13.4% in high humidity.

## 5. Conclusions

Our findings confirmed handwashing with non-antimicrobial soap reduced the risks associated with indirect transmission of *E. coli* infection from contact with fomites during food preparation by approximately 5-fold compared to the risk calculation with no handwashing. Further, in these exposure scenarios, washing with antimicrobial soap reduced the risk of *E. coli* infection from touch transfer by an average of about 40-fold compared with no handwashing. The antimicrobial soaps ranged from 3 to 32 times more effective than the non-antimicrobial soap, depending on the specific exposure scenario.

## Figures and Tables

**Table 1:** Parameters and their distribution used in exposure assessment.

**a.** Transfer of *E. coli* to and between fomites.						
***E. coli* Concentration on Primary Surface (Fomite)**			**Transfer to Secondary Surface**			
**Primary Surface**	**Input Values (Range) ^a^**	**Distribution**	**Secondary Surface**	**Input Values (range) ^a^**	**Distribution**	**Source(s)**
	**(log_10_ CFU/sq. cm)**			**(%)**		
**Meat Preparation Surfaces**						
Cutting board ^b^	5.80	Uniform	Cutting board-to-meat	32.57	Normal	[21]
(high dose)	(5.54, 6.06)		(high dose)	(0.86, 93.41)		
Cutting board	3.73	Uniform	Cutting board-to-meat	55.91	Normal	[21]
(low dose)	(3.63, 3.82)		(low dose)	(23.17, 97.31)		
Knife ^c^	6.08	Uniform	Knife-to-meat	37.98	Normal	[21]
(high dose)	(6.01, 6.15)		(high dose)	(24.15, 37.98)		
Knife	4.21	Uniform	Knife-to-meat	50.42	Normal	[21]
(low dose)	(3.53, 4.89)		(low dose)	(12.82, 109.64)		
Meat	6.23	Uniform	Meat-to-cutting board	53.01	Normal	[21]
(high dose)	(6.06, 6.39)		(high dose)	(42.03, 74.18)		
Meat	4.33	Uniform	Meat-to-cutting board	62.94	Normal	[21]
(low dose)	(3.69, 4.97)		(low dose)	(34.28, 89.75)		
Meat	6.33	Uniform	Meat-to-knife	49.09	Normal	[21]
(high dose)	(6.15, 6.5)		(high dose)	(37.75, 49.09)		
Meat	4.03	Uniform	Meat-to-knife	56.17	Normal	[21]
(low dose)	(3.87, 4.18)		(low dose)	(21.86, 105.69)		
**Typical Kitchen Surfaces**						
Countertop ^d^	2.36	Triangular				[22]
	(0.78, 3.00)					
Hand towel	1.79	Triangular				[23]
	(0.13, 3.86)					
Dish cloth	1.75	Triangular				[24]
	(0.12, 4.25)					
Kitchen sponge	4.00	Triangular				[25]
	(3.00, 6.00)					
**b.** Transfer from fomites to fingertip.						
		**Input Values (Range) ^a^**		**Distribution**		**Source(s)**
		**(%)**				
Meat-to-fingertip ^e^		34.31		Lognormal		[26]
		(34.31, 166.37)				
Cutting board-to-fingertip		53.3		Triangular		[27]
		(30.40, 98.00)				
Knife-to-fingertip		54.1		Triangular		[27]
		(29.40, 99.00)				
Countertop-to-fingertip		36.5		Triangular		[27]
		(0.30, 100.00)				
Hand towel-to-fingertip ^f^		13.4		Triangular		[27]
		(2.60, 33.30)				
Dish cloth-to-fingertip ^f^		13.4		Triangular		[27]
		(2.60, 33.30)				
Kitchen sponge-to-fingertip		13.4		Triangular		[27]
		(2.60, 33.30)				
**c.** Bacterial survival after hand washing and transfer to lip.						
		**Input Values (range) ^a^**		**Distribution**		**Source(s)**
		**(%)**				
Survival of *E. coli* on hand after control wash		13.49		Normal		g
		(5.04, 36.06)				
Survival of *E. coli* on hand after wash with antimicrobial #1		1.12		Normal		g
		(0.16, 7.80)				
Survival of *E. coli* on hand after wash with antimicrobial #2		0.72		Normal		g
		(0.04, 11.96)				
Transfer from fingertip-to-lip		28.6		Lognormal		[28]
		(28.60, 63.07)				

a = For uniform distribution, mean (low, high) values are presented. For normal distribution, mean (5%, 95%) values are presented. For lognormal distribution, mean (mean, standard deviation) values are presented. For triangular distribution, likeliest (minimum, maximum) values are presented. b = Input values measured for HDPE were used. c = Input values measured for stainless steel were used. d = Input values measured for granite were used. e = *Enterobacter aerogenes* B199A was used as a surrogate microorganism. f = Input values measured for 100% cotton were used. g = Original data described in Methods Section 2.1. The input values given in Table 1 were used in Monte Carlo simulation. This was a single-touch scenario. Survival of *E. coli* at the fingertip was estimated under four scenarios: No hand washing, washing with a control (non-antimicrobial) bar soap, and washing with two different antimicrobial bars designated #1 and #2. Note that the study did not consider the natural growth or decay of *E. coli*, the timeframe of the contact, or the frequency of touching.

**Table 2:** Experimental conditions for the *E. coli* dose-response experiment for the selected models found in QMRAWiki [29].

	Host Type	Agent Strain	Route	# of Doses	Dose Units	Response	Best fit Model	Optimized Parameter(s)	ID50
Recommended best fit model	Human	EIEC 1624	Oral (in milk)	3	CFU	Positive stool isolation	beta-Poisson	α = 1.55×10^−1^ N50 = 2.11 × 10^6^	2.11 × 10^6^
High infectious dose model	Human	ETEC B7A	Oral (in milk)	11	CFU	Mild to severe diarrhea	beta-Poisson	α = 2.06 × 10^−1^ N50 = 1.28 × 10^8^	1.28 × 10^8^
Low infectious dose model	Human	ETEC B7A	Oral (in milk)	7	CFU	Positive stool isolation	beta-Poisson	α = 3.75 × 10^−1^ N50 = 1.78 × 10^5^	1.78 × 10^5^

**Table 3:** Exposure comparison with different hand washing practices.

**a.** Potential oral dose from different exposure protocols				
	**Median CFU** **(5%, 95%)**			
	**No Washing**	**Control Bar**	**Antimicrobial #1**	**Antimicrobial #2**
**Transfer from Meat Preparation Surfaces**				
Cutting board-to-meat	1.9 × 10^3^	3.5 × 10^2^	5.9 × 10^1^	9.0 × 10^1^
(high dose)	(0.0 × 10^0^, 9.7 × 10^4^)	(0.0 × 10^0^, 2.0 × 10^4^)	(0.0 × 10^0^, 3.8 × 10^3^)	(0.0 × 10^0^, 5.5 × 10^3^)
Cutting board-to-meat	2.4 × 10^1^	4.3 × 10^0^	7.6 × 10^−1^	1.1 × 10^0^
(low dose)	(5.7 × 10^−1^, 9.9 × 10^2^)	(6.3 × 10^−2^, 2.1 × 10^2^)	(2.9 × 10^−4^, 4.0 × 10^1^)	(0.0 × 10^0^, 6.0 × 10^1^)
Knife-to-meat	5.8 × 10^3^	1.0 × 10^3^	1.8 × 10^2^	2.6 × 10^2^
(high dose)	(1.4 × 10^2^, 2.4 × 10^5^)	(1.6 × 10^1^, 5.0 × 10^4^)	(2.9 × 10^−2^, 9.1 × 10^3^)	(0.0 × 10^0^, 1.4 × 10^4^)
Knife-to-meat	7.5 × 10^1^	1.3 × 10^1^	2.3 × 10^0^	3.5 × 10^0^
(low dose)	(8.6 × 10^−1^, 4.6 × 10^3^)	(7.7 × 10^−2^, 9.1 × 10^2^)	(0.0 × 10^0^, 1.8 × 10^2^)	(0.0 × 10^0^, 2.5 × 10^2^)
Meat-to-cutting board	6.7 × 10^4^	1.2 × 10^4^	2.2 × 10^3^	3.3 × 10^3^
(high dose)	(7.2 × 10^3^, 6.6 × 10^5^)	(8.3 × 10^2^, 1.4 × 10^5^)	(3.5 × 10^1^, 2.8 × 10^4^)	(0.0 × 10^0^, 4.1 × 10^4^)
Meat-to-cutting board	9.1 × 10^2^	1.6 × 10^2^	3.0 × 10^1^	4.4 × 10^1^
(low dose)	(8.0 × 10^1^, 1.2 × 10^4^)	(8.2 × 10^0^, 2.5 × 10^3^)	(4.9 × 10^−1^, 5.1 × 10^2^)	(0.0 × 10^0^, 7.7 × 10^2^)
Meat-to-knife	1.8 × 10^5^	2.4 × 10^4^	2.0 × 10^3^	1.3 × 10^3^
(high dose)	(1.2 × 10^5^, 2.6 × 10^5^)	(1.6 × 10^4^, 3.5 × 10^4^)	(1.3 × 10^3^, 2.9 × 10^3^)	(8.5 × 10^2^, 1.9 × 10^3^)
Meat-to-knife	1.0 × 10^3^	1.4 × 10^2^	1.2 × 10^1^	7.3 × 10^0^
(low dose)	(6.6 × 10^2^, 1.5 × 10^3^)	(9.0 × 10^1^, 2.0 × 10^2^)	(7.5 × 10^0^, 1.7 × 10^1^	(4.8 × 10^0^, 1.1 × 10^1^)
**Transfer from Typical Kitchen Surfaces**				
Countertop	9.9 × 10^−1^	1.8 × 10^−1^	3.3 × 10^−2^	4.9 × 10^−2^
	(8.0 × 10^−2^, 2.0 × 10^1^)	(9.8 × 10^−3^, 4.2 × 10^0^)	(7.8 × 10^−4^, 7.9 × 10^−1^)	(1.6 × 10^−5^, 1.2 × 10^0^)
Hand towel	2.5 × 10^−1^	4.6 × 10^−2^	3.2 × 10^−4^	0.0 × 10^0^
	(3.5 × 10^−2^, 2.3 × 10^0^)	(4.2 × 10^−3^, 4.8 × 10^−1^)	(0.0 × 10^0^, 8.5 × 10^−2^)	(0.0 × 10^0^, 1.2 × 10^−1^)
Dish cloth	2.6 × 10^−1^	4.7 × 10^−2^	8.7 × 10^−3^	1.3 × 10^−2^
	(3.4 × 10^−2^, 2.5 × 10^0^)	(4.1 × 10^−3^, 5.4 × 10^−1^)	(1.7 × 10^−4^, 1.1 × 10^−1^	(0.0 × 10^0^, 1.6 × 10^−1^)
Kitchen sponge	6.1 × 10^−1^	1.1 × 10^−1^	2.1 × 10^−2^	3.0 × 10^−2^
	(6.8 × 10^−2^, 6.9 × 10^0^)	(8.3 × 10^−3^, 1.4 × 10^0^)	(3.7 × 10^−4^, 2.9 × 10^−1^)	(2.0 × 10^−4^, 4.4 × 10^−1^)
**b.** Exposure reduction factor ^a^ compared to “no washing”.				
		**Control Bar**	**Antimicrobial #1**	**Antimicrobial #2**
**Transfer from Meat Preparation Surfaces**				
Cutting board-to-meat (high dose)		5.4	32	21
Cutting board-to-meat (low dose)		5.6	32	22
Knife-to-meat (high dose)		5.8	32	22
Knife-to-meat (low dose)		5.8	33	21
Meat-to-cutting board (high dose)		5.6	30	20
Meat-to-cutting board (low dose)		5.7	30	21
Meat-to-knife (high dose)		7.5	90	138
Meat-to-knife (low dose)		7.1	83	137
**Transfer from Typical Kitchen Surfaces**				
Countertop		5.5	30	20
Hand towel		5.4	781	>781 ^b^
Dish cloth		5.5	30	20
Kitchen sponge		5.5	29	20

a = Determined by dividing the median exposure dose from “no washing” by the median exposure dose after washing with one of three soaps. b = Cannot be calculated. The median predicted exposure dose was 0.0 × 10^0^ CFU.

**Table 4:** Risk comparison with different hand washing practices.

**a.** Estimated risk from different exposure protocols calculated using three dose models.												
	**Risk with Recommended Dose Response Model**				**Risk with High Infectious Dose Model**				**Risk with Low Infectious Dose Model**			
	**Median (5%, 95%)**				**Median (5%, 95%)**				**Median (5%, 95%)**			
	**No Washing**	**Control Bar**	**Antimicrobial #1**	**Antimicrobial #2**	**No Washing**	**Control Bar**	**Antimicrobial #1**	**Antimicrobial #2**	**No Washing**	**Control Bar**	**Antimicrobial #1**	**Antimicrobial #2**
**Transfer from Meat Preparation Surfaces**												
Cutting board-to-meat (high dose)	1.20 × 10^−2^	2.20 × 10^−3^	3.80 × 10^−4^	5.70 × 10^−4^	8.70 × 10^−5^	1.60 × 10^−5^	2.70 × 10^−6^	4.10 × 10^−6^	2.10 × 10^−2^	3.90 × 10^−3^	6.70 × 10^−4^	1.00 × 10^−3^
	(0.0 × 10^0^, 2.2 × 10^−1^)	(0.0 × 10^0^, 8.8 × 10^−2^)	(0.0 × 10^0^, 2.2 × 10^−2^)	(0.0 × 10^0^, 3.1 × 10^−2^)	(0.0 × 10^0^, 4.3 × 10^−3^)	(0.0 × 10^0^, 8.8 × 10^−4^)	(0.0 × 10^0^, 1.7 × 10^−4^)	(0.0 × 10^0^, 2.5 × 10^−4^)	(0.0 × 10^0^, 4.0 × 10^−1^)	(0.0 × 10^0^, 1.6 × 10^−1^)	(0.0 × 10^0^, 4.0 × 10^−2^)	(0.0 × 10^0^, 5.6 × 10^−2^)
Cutting board-to-meat (low dose)	1.60 × 10^−4^	2.70 × 10^−5^	4.80 × 10^−6^	7.20 × 10^−6^	1.10 × 10^−6^	1.90 × 10^−7^	3.40 × 10^−8^	5.10 × 10^−8^	2.70 × 10^−4^	4.80 × 10^−5^	8.50 × 10^−6^	1.30 × 10^−5^
	(3.6 × 10^−6^, 6.1 × 10^−3^)	(4.0 × 10^−7^, 1.3 × 10^−3^)	(1.8 × 10^−9^, 2.6 × 10^−4^)	(0.0 × 10^0^, 3.8 × 10^−4^)	(2.6 × 10^−8^, 4.4 × 10^−5^)	(2.8 × 10^−9^, 9.6 × 10^−6^)	(1.3 × 10^−11^, 1.8 × 10^−6^)	(0.0 × 10^0^, 2.7 × 10^−6^)	(6.5 × 10^−6^, 1.1 × 10^−2^)	(7.1 × 10^−7^, 2.4 × 10^−3^)	(3.2 × 10^−9^, 4.5 × 10^−4^)	(0.0 × 10^0^, 6.8 × 10^−4^)
Knife-to-meat (high dose)	3.20 × 10^−2^	6.50 × 10^−3^	1.10 × 10^−3^	1.70 × 10^−3^	2.60 × 10^−4^	4.70 × 10^−5^	7.90 × 10^−6^	1.20 × 10^−5^	5.80 × 10^−2^	1.20 × 10^−2^	2.00 × 10^−3^	3.00 × 10^−3^
	(9.2 × 10^−4^, 3.1 × 10^−1^)	(9.9 × 10^−5^, 1.6 × 10^−1^)	(1.8 × 10^−7^, 4.8 × 10^−2^)	(0.0 × 10^0^, 6.9 × 10^−2^)	(6.5 × 10^−6^, 1.0 × 10^−2^)	(7.0 × 10^−7^, 2.2 × 10^−3^)	(1.3 × 10^−9^, 4.1 × 10^−4^)	(0.0 × 10^0^, 6.4 × 10^−4^)	(1.6 × 10^−3^, 5.4 × 10^−1^)	(1.8 × 10^−4^, 2.9 × 10^−1^)	(3.3 × 10^−7^, 8.6 × 10^−2^)	(0.0 × 10^0^, 1.3 × 10^−1^)
Knife-to-meat (low dose)	4.70 × 10^−4^	8.40 × 10^−5^	1.50 × 10^−5^	2.20 × 10^−5^	3.40 × 10^−6^	5.90 × 10^−7^	1.00 × 10^−7^	1.60 × 10^−7^	8.40 × 10^−4^	1.50 × 10^−4^	2.60 × 10^−5^	3.90 × 10^−5^
	(5.5 × 10^−6^, 2.6 × 10^−2^)	(4.9 × 10^−7^, 5.6 × 10^−3^)	(0.0 × 10^0^, 1.1 × 10^−3^)	(0.0 × 10^0^, 1.6 × 10^−3^)	(3.9 × 10^−8^, 2.1 × 10^−4^)	(3.5 × 10^−9^, 4.1 × 10^−5^)	(0.0 × 10^0^, 7.9 × 10^−6^)	(0.0 × 10^0^, 1.1 × 10^−5^)	(9.7 × 10^−6^, 4.7 × 10^−2^)	(8.7 × 10^−7^, 1.0 × 10^−2^)	(0.0 × 10^0^, 2.0 × 10^−3^)	(0.0 × 10^0^, 2.8 × 10^−3^)
Meat-to-cutting board (high dose)	1.90 × 10^−1^	6.00 × 10^−2^	1.40 × 10^−2^	2.00 × 10^−2^	3.00 × 10^−3^	5.40 × 10^−4^	1.00 × 10^−4^	1.50 × 10^−4^	3.40 × 10^−1^	1.10 × 10^−1^	2.40 × 10^−2^	3.50 × 10^−2^
	(3.9 × 10^−2^, 4.0 × 10^−1^)	(5.2 × 10^−3^, 2.6 × 10^−1^)	(2.2 × 10^−4^, 1.1 × 10^−1^)	(0.0 × 10^0^, 1.4 × 10^−1^)	(3.2 × 10^−4^, 2.7 × 10^−2^)	(3.5 × 10^−5^, 6.3 × 10^−3^)	(1.6 × 10^−6^, 1.3 × 10^−3^)	(0.0 × 10^0^, 1.8 × 10^−3^)	(7.1 × 10^−2^, 6.8 × 10^−1^)	(9.2 × 10^−3^, 4.7 × 10^−1^)	(4.0 × 10^−4^, 2.1 × 10^−1^)	(0.0 × 10^0^, 2.6 × 10^−1^)
Meat-to-cutting board (low dose)	5.70 × 10^−3^	1.00 × 10^−3^	1.90 × 10^−4^	2.80 × 10^−4^	4.10 × 10^−5^	7.30 × 10^−6^	1.40 × 10^−6^	2.00 × 10^−6^	1.00 × 10^−2^	1.80 × 10^−3^	3.40 × 10^−4^	5.00 × 10^−4^
	(5.1 × 10^−4^, 6.1 × 10^−2^)	(5.2 × 10^−5^, 1.5 × 10^−2^)	(3.1 × 10^−6^, 3.2 × 10^−3^)	(0.0 × 10^0^, 4.8 × 10^−3^)	(3.6 × 10^−6^, 5.5 × 10^−4^)	(3.7 × 10^−7^, 1.1 × 10^−4^)	(2.2 × 10^−8^, 2.3 × 10^−5^)	(0.0 × 10^0^, 3.5 × 10^−5^)	(9.0 × 10^−4^, 1.1 × 10^−1^)	(9.3 × 10^−5^, 2.7 × 10^−2^)	(5.5 × 10^−6^, 5.7 × 10^−3^)	(0.0 × 10^0^, 8.6 × 10^−3^)
Meat-to-knife (high dose)	2.80 × 10^−1^	1.00 × 10^−1^	1.20 × 10^−2^	7.90 × 10^−3^	7.80 × 10^−3^	1.10 × 10^−3^	9.00 × 10^−5^	5.80 × 10^−5^	5.00 × 10^−1^	1.90 × 10^−1^	2.20 × 10^−2^	1.40 × 10^−2^
	(2.4 × 10^−1^, 3.2 × 10^−1^)	(7.5 × 10^−2^, 1.3 × 10^−1^)	(8.2 × 10^−3^, 1.7 × 10^−2^)	(5.3 × 10^−3^, 1.1 × 10^−2^)	(5.2 × 10^−3^, 1.1 × 10^−2^)	(7.1 × 10^−4^, 1.6 × 10^−3^)	(6.0 × 10^−5^, 1.3 × 10^−4^)	(3.8 × 10^−5^, 8.4 × 10^−5^)	(4.3 × 10^−1^, 5.6 × 10^−1^)	(1.4 × 10^−1^, 2.4 × 10^−1^)	(1.5 × 10^−2^, 3.1 × 10^−2^)	(9.4 × 10^−3^, 2.0 × 10^−2^)
Meat-to-knife (low dose)	6.30 × 10^−3^	8.70 × 10^−4^	7.30 × 10^−5^	4.70 × 10^−5^	4.60 × 10^−5^	6.20 × 10^−6^	5.20 × 10^−7^	3.30 × 10^−7^	1.10 × 10^−2^	1.60 × 10^−3^	1.30 × 10^−4^	3.80 × 10^−5^
	(4.2 × 10^−3^, 9.1 × 10^−3^)	(5.7 × 10^−4^, 1.3 × 10^−3^)	(4.7 × 10^−5^, 1.1 × 10^−4^)	(3.0 × 10^−5^, 6.8 × 10^−5^)	(3.0 × 10^−5^, 6.7 × 10^−5^)	(4.0 × 10^−6^, 9.0 × 10^−6^)	(3.4 × 10^−7^, 7.5 × 10^−7^)	(2.1 × 10^−7^, 4.8 × 10^−7^)	(7.4 × 10^−3^, 1.6 × 10^−2^)	(1.0 × 10^−3^, 2.2 × 10^−3^)	(8.4 × 10^−5^, 1.9 × 10^−4^)	(5.4 × 10^−5^, 1.2 × 10^−4^)
**Transfer from Typical Kitchen Surfaces**												
Countertop	6.30 × 10^−6^	1.20 × 10^−6^	2.10 × 10^−7^	3.10 × 10^−7^	4.50 × 10^−8^	2.80 × 10^−9^	1.50 × 10^−9^	2.20 × 10^−9^	1.10 × 10^−5^	2.10 × 10^−6^	3.70 × 10^−7^	5.50 × 10^−7^
	(5.1 × 10^−7^, 1.3 × 10^−4^)	(6.2 × 10^−8^, 2.7 × 10^−5^)	(5.0 × 10^−9^, 5.0 × 10^−6^)	(1.0 × 10^−10^, 7.8 × 10^−6^)	(3.6 × 10^−9^, 9.0 × 10^−7^)	(4.4 × 10^−10^, 1.9 × 10^−7^)	(3.5 × 10^−11^, 3.6 × 10^−8^)	(7.1 × 10^−13^, 5.6 × 10^−8^)	(9.0 × 10^−7^, 2.3 × 10^−4^)	(1.1 × 10^−7^, 4.8 × 10^−5^)	(8.8 × 10^−9^, 8.9 × 10^−6^)	(1.8 × 10^−10^, 1.4 × 10^−5^)
Hand towel	1.60 × 10^−6^	2.90 × 10^−7^	2.00 × 10^−9^	0.00 × 10^0^	1.10 × 10^−8^	2.10 × 10^−9^	1.40 × 10^−11^	0.00 × 10^0^	2.80 × 10^−6^	5.20 × 10^−7^	3.60 × 10^−9^	0.00 × 10^0^
	(2.2 × 10^−7^, 1.5 × 10^−5^)	(2.6 × 10^−8^, 3.1 × 10^−6^)	(0.0 × 10^0^, 5.4 × 10^−7^)	(0.0 × 10^0^, 7.8 × 10^−7^)	(1.6 × 10^−9^, 1.1 × 10^−7^)	(1.9 × 10^−10^, 2.2 × 10^−8^)	(0.0 × 10^0^, 3.8 × 10^−9^)	(0.0 × 10^0^, 5.5 × 10^−9^)	(3.9 × 10^−7^, 2.6 × 10^−5^)	(4.7 × 10^−8^, 5.4 × 10^−6^)	(0.0 × 10^0^, 9.5 × 10^−7^)	(0.0 × 10^0^, 1.4 × 10^−6^)
Dish cloth	1.60 × 10^−6^	3.00 × 10^−7^	5.50 × 10^−8^	8.10 × 10^−8^	1.20 × 10^−8^	2.10 × 10^−9^	3.90 × 10^−10^	5.70 × 10^−10^	2.90 × 10^−6^	5.30 × 10^−7^	9.80 × 10^−8^	1.40 × 10^−7^
	(2.1 × 10^−7^, 1.6 × 10^−5^)	(2.6 × 10^−8^, 3.4 × 10^−6^)	(1.1 × 10^−9^, 6.9 × 10^−7^)	(0.0 × 10^0^, 1.0 × 10^−6^)	(1.5 × 10^−9^, 1.1 × 10^−7^)	(1.8 × 10^−10^, 2.4 × 10^−8^)	(7.7 × 10^−12^, 4.9 × 10^−9^)	(0.0 × 10^0^, 7.3 × 10^−9^)	(3.8 × 10^−7^, 2.9 × 10^−5^)	(4.6 × 10^−8^, 6.0 × 10^−6^)	(1.9 × 10^−9^, 1.2 × 10^−6^)	(0.0 × 10^0^, 1.8 × 10^−6^)
Kitchen sponge	3.90 × 10^−6^	7.30 × 10^−7^	1.30 × 10^−7^	1.90 × 10^−7^	2.80 × 10^−8^	5.10 × 10^−9^	9.30 × 10^−10^	1.40 × 10^−9^	6.90 × 10^−6^	1.30 × 10^−6^	2.30 × 10^−7^	3.40 × 10^−7^
	(4.3 × 10^−7^, 4.4 × 10^−5^)	(5.3 × 10^−8^, 9.1 × 10^−6^)	(2.3 × 10^−9^, 1.9 × 10^−6^)	(1.3 × 10^−9^, 2.8 × 10^−6^)	(3.1 × 10^−9^, 3.1 × 10^−7^)	(3.7 × 10^−10^, 6.5 × 10^−8^)	(1.6 × 10^−11^, 1.3 × 10^−8^)	(9.1 × 10^−12^, 2.0 × 10^−8^)	(7.7 × 10^−7^, 7.8 × 10^−5^)	(9.4 × 10^−8^, 1.6 × 10^−5^)	(4.1 × 10^−9^, 3.3 × 10^−6^)	(2.3 × 10^−9^, 4.9 × 10^−6^)
**b**. Risk reduction factor ^a^ with different hand washing protocols compared to “no washing”.												
				**Control Bar**			**Antimicrobial #1**			**Antimicrobial #2**		
**Transfer from Meat Preparation Surfaces**												
Cutting board-to-meat (high dose)				5.5			32			21		
Cutting board-to-meat (low dose)				5.9			33			22		
Knife-to-meat (high dose)				4.9			29			19		
Knife-to-meat (low dose)				5.6			31			21		
Meat-to-cutting board (high dose)				3.2			14			10		
Meat-to-cutting board (low dose)				5.7			30			20		
Meat-to-knife (high dose)				2.8			23			35		
Meat-to-knife (low dose)				7.2			86			134		
**Transfer from Typical Kitchen Surfaces**												
Kitchen surface				5.3			30			20		
Hand towel				5.5			800			>800		
Dish cloth				5.3			29			20		
Kitchen sponge				5.3			30			21		

a = Determined by comparing the median estimated risk from “no washing” to the median risk after washing with each of the three bar soaps. Calculations using the recommended best fit model are shown. b = Could not be calculated since the median estimated risk was 0.0 × 10^0^.
